# Metabolism in the progression and metastasis of brain tumors

**DOI:** 10.1016/j.canlet.2022.215713

**Published:** 2022-05-02

**Authors:** Abhishek Tyagi, Shih-Ying Wu, Kounosuke Watabe

**Affiliations:** Department of Cancer Biology, Wake Forest University School of Medicine, Winston-Salem, NC, 27157, USA

**Keywords:** Brain tumor, Brain metastasis, Metabolic reprogramming, Cancer, Immune cell metabolism

## Abstract

Malignant brain tumors and metastases pose significant health problems and cause substantial morbidity and mortality in children and adults. Based on epidemiological evidence, gliomas comprise 30% and 80% of primary brain tumors and malignant tumors, respectively. Brain metastases affect 15–30% of cancer patients, particularly primary tumors of the lung, breast, colon, and kidney, and melanoma. Despite advancements in multimodal molecular targeted therapy and immunotherapy that do not ensure long-term treatment, malignant brain tumors and metastases contribute significantly to cancer related mortality. Recent studies have shown that metastatic cancer cells possess distinct metabolic traits to adapt and survive in new environment that differs significantly from the primary site in both nutrient composition and availability. As metabolic regulation lies at the intersection of many research areas, concerted efforts to understand the metabolic mechanism(s) driving malignant brain tumors and metastases may reveal novel therapeutic targets to prevent or reduce metastasis and predict biomarkers for the treatment of this aggressive disease. This review focuses on various aspects of metabolic signaling, interface between metabolic regulators and cellular processes, and implications of their dysregulation in the context of brain tumors and metastases.

## 1. Introduction

**Fig. 1.**
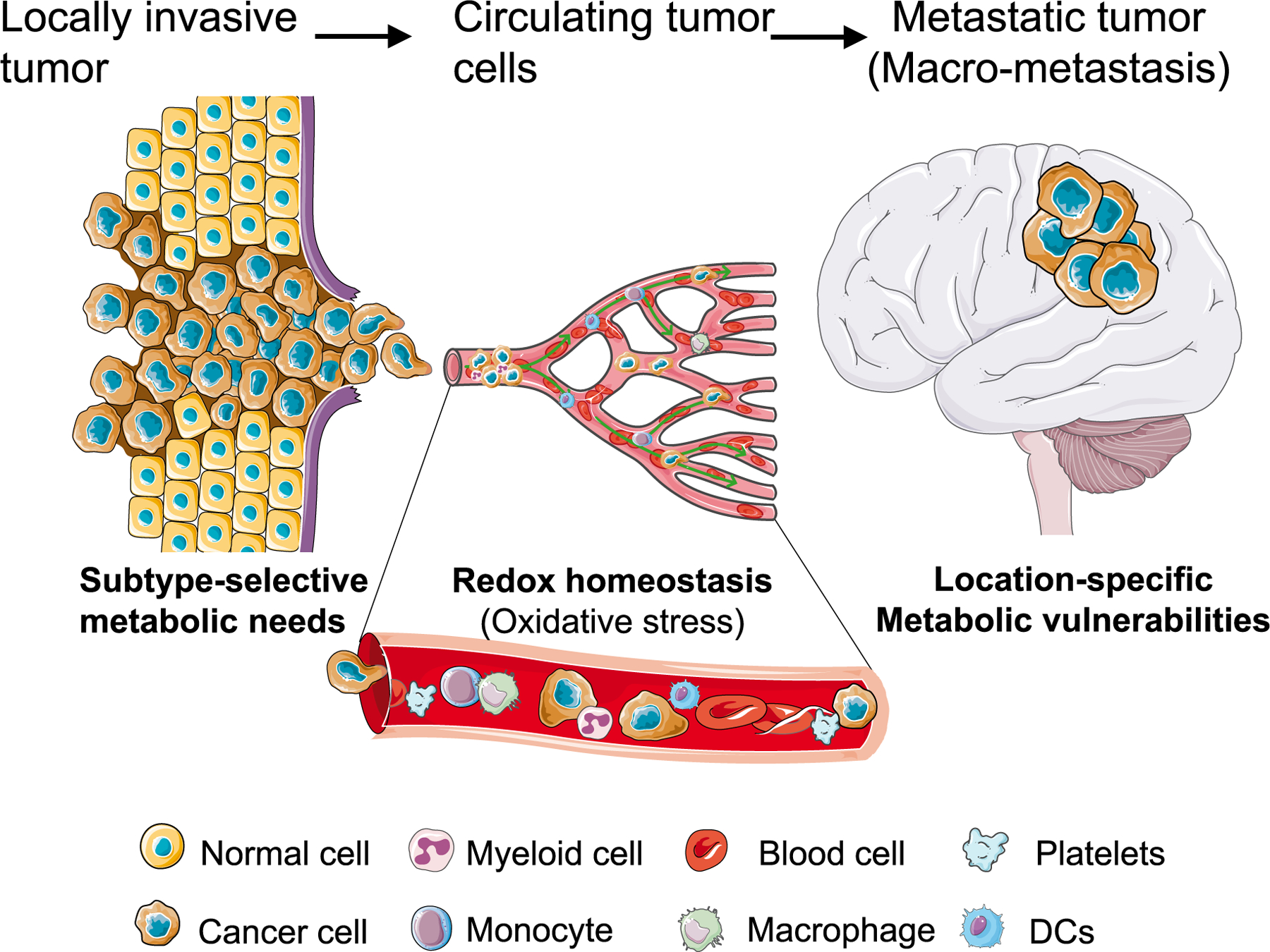
Metabolism and its impact on cancer progression.

Malignant brain tumors and metastases are generally fatal because of their extremely poor prognosis and significant morbidity and mortality in children and adults. Glioma, the most common primary brain tumor [1] and brain metastases from other primary tumors account for considerable proportion of central nervous system (CNS) neoplasms [2]. Approximately 15–30% of patients with metastatic lung, breast, melanoma, renal and colorectal malignancies develop brain metastases [2,3]. Despite the use of advanced therapies such as surgery, radiation, chemotherapy, and immunotherapy, only a small percentage of patients with malignant brain tumor and metastasis survive less than two years after the initial diagnosis [2,4]. Crosstalk between cancer cells and non-neoplastic cells in the brain tumor microenvironment (TME) appears to play a crucial role in tumor progression and therapy resistance, which can be directly targeted in modulating brain tumors and metastases that account for 90% of cancer-related mortality [5]. Furthermore, the phenotypic complexity observed during metastasis strongly suggests that cancer cells overcome certain biological challenges to establish metastatic colonization at distant sites [6]. During this process, metabolic plasticity, which regulates dynamic energy sources and metabolic intermediates, plays a key role in modifying the intrinsic and extrinsic factors contributing to metastatic potential of tumors in distant organs [7]. Despite extensive research on the role of metabolic reprogramming in promoting primary tumor growth, its role in facilitating metastasis remains unknown. Furthermore, the significance of metabolic changes that enable cancer cells to metastasize, and establish relationship with their local environment, warrants further investigation for better understanding (Fig. 1). Therefore, in the era of precision medicine, focusing on targeted metabolism, either in immune cells or tumor cells, will open new avenues for innovative possibilities by providing a better understanding of how to build a therapeutic window for intervention in brain tumors and metastasis.

## 2. Metabolism of brain tumors

Brain tumors constitute 2% of all adult tumors and 23% of primary childhood tumors [8]. Majority of malignant primary brain tumors and metastases are located in the cerebral cortex region of the CNS, with the frontal lobe having the highest incidence [9]. Gliomas are primary brain tumors that arise from the intrinsic components of the CNS and account for approximately 80% of malignant brain tumors [4]. They are often classified as biologically aggressive owing to their histological characteristics and resemblance to differentiated glial lineages, such as astrocytes, oligodendrocytes, and ependymal cells [5]. Glioblastoma (GBM), the highest grade and most lethal brain tumor is the most common form of glioma in adults, and has a median survival of less than 15 months when treated with standard surgical interventions and accessible targeted clinical therapies [10].

**Fig. 2.**
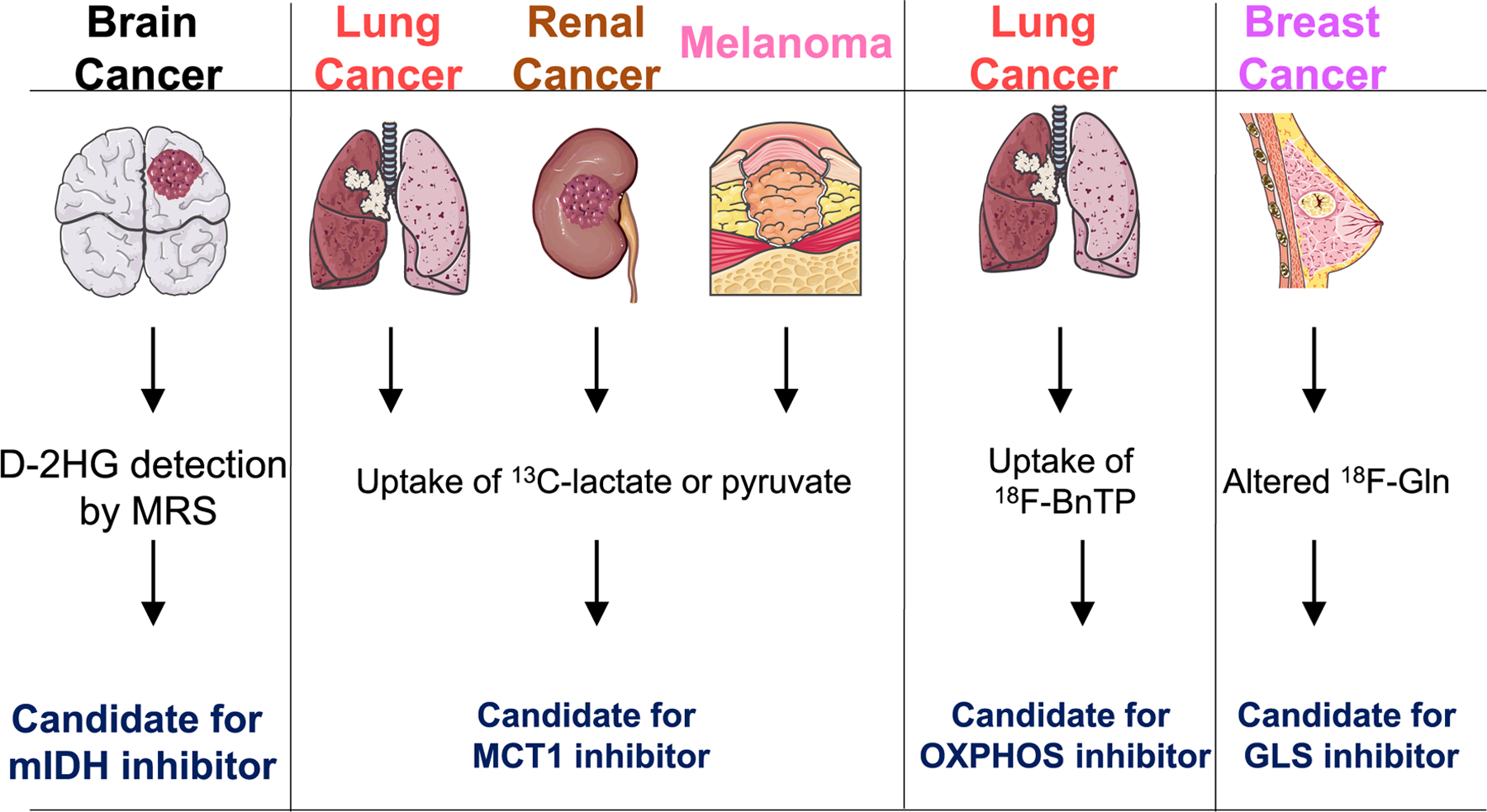
Prospects for assessing *In Vivo* tumor metabolism with metabolic targeting.

**Fig. 3.**
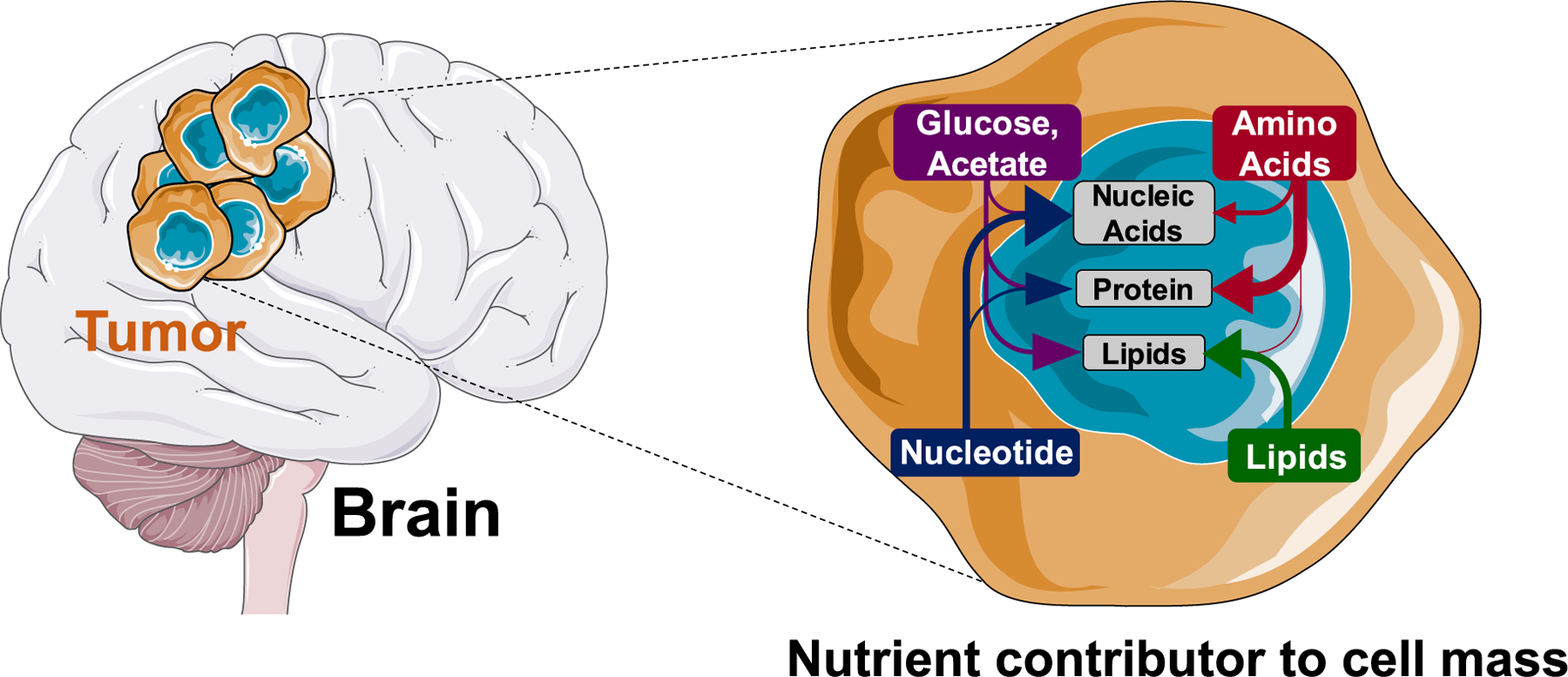
Potential Metabolic Vulnerabilities for Drug Identification in Brain tumor.

The brain is a highly metabolically active organ that primarily uses glucose as an energy substrate. Accumulating evidence suggest the importance of ketone bodies as key alternative metabolic fuel to support the energy needs of the brain during development [11] and in glioma during glucose starvation [12]. Furthermore, diverse cell types in the brain interact and influence each other’s nutrient uptake, and thus maintain metabolic homeostasis in the brain. Astrocytes, for example, depend on neuron-derived glutamate to function during metabolic cooperation, whereas neurons, although previously reported to rely on astrocytic lactate, primarily depend on glucose during neuronal stimulation [13,14]. Furthermore, brain tumors develop in a complex, frequent hypoxic metabolic environment [15], which alters the repertoire of metabolic options that cancer cells embark on to drive aggressive tumor growth. Interestingly, recent technological advances in *in vivo* metabolic analysis tools, including isotope-labeled metabolite tracing and non-invasive metabolic imaging (Fig. 2), have enabled in-depth analysis of metabolic fluxes in brain tumor and diverse preclinical and clinical samples. This will eventually help in developing a rational approach for new strategies in improving cancer diagnosis, prognosis, and therapeutic interventions. The potential implications of widely reported energy metabolism in brain tumors (Fig. 3) are discussed in detail.

### 2.1. Glucose and acetate

Glucose and acetate are the most abundant nutrients in the brain, and GBM tumor cells rapidly assimilate them as bioenergetic substrates [16]. Like other cancer cell types, GBM cells have been reported to utilize both glycolysis and oxidative phosphorylation (OXPHOS) during aggressive tumor growth [16]. By employing an isotope-labeled metabolite tracing approach in GBM pre-clinical models and human clinical samples of brain tumors and metastases, a recent study identified acetate from the microenvironment as a critical bioenergetic substrate responsible for tumor progression. This suggests that acetate, by improving metabolic flexibility, may help cancer cells survive in the brain microenvironment [17]. Cancer cells can also oxidize and maintain an intracellular pool of acetate by upregulating acetyl-CoA synthetase 2 (ACSS2) in the tricarboxylic acid (TCA) cycle [17], which converts acetate into acetyl-CoA [18]. This suggests that during the growth and progression of brain tumor and metastases, cancer cells have high biosynthetic and bioenergetic demand to adapt to foreign environments.

### 2.2. Amino acids

Glutamine, an essential amino acid, is a carbon and nitrogen source for cancer cells, allowing them to utilize glucose-derived carbon and TCA cycle intermediates in the synthetic pathway, as observed in GBM [19]. This implies that the cancer cell metabolism is optimized to maximize nutrient availability and absorption into biomass, causing tumor growth. Interestingly, human GBM xenografts, compared to surrounding normal brain tissue, showed an increased pool of glutamine, and their ability to metabolize it for tumor cell proliferation and survival [16]. Magnetic resonance spectroscopy (MRS) revealed elevated intra-tumoral glutamine levels compared to glutamine levels in contralateral normal brain tissues in GBM patients [20]. Moreover, glutamine-starved GBM cells showed upregulation of glutamine synthetase (GS) that promotes the conversion of glutamate into glutamine [21]; this enzyme has been found to be highly expressed in GBM clinical samples [21], implying that tumor cells utilize this complementary pathway to obtain glutamine required for their growth. Furthermore, gliomas with mutated isocitrate dehydrogenase (IDH1 and IDH2) showed elevated levels of oncometabolite D-2-hydroxyglutarate derived from glutamine *via* glutamate and α-KG conversion, demonstrating the importance of glutamine metabolism in the malignant progression of gliomas [22]. Future research using fluoro-analogs of glutamine for *in vivo* analyses in pre-clinical models by positron emission tomography (PET) imaging [23], along with clinical correlation will be critical in understanding how cellular glutamine pools are maintained and utilized in driving glioma metabolism.

### 2.3. Lipids

Approximately 50% of the brain’s dry weight is composed of lipids, which are structural and functional components of the brain [24]. Brain is the most cholesterol-rich organ; it contains 20% of total cholesterol of the body [24]. Because circulating cholesterol in lipoproteins cannot cross the blood-brain barrier (BBB) efficiently, almost all cholesterol present in the brain is formed by *de novo* synthesis by astrocytes, and used as a high-energy source [25]. Importantly, brain tumor cells consume significantly more extracellular cholesterol than normal cells [26]. Furthermore, brain tumor cells can use acetyl-CoA derived from glucose, acetate, and TCA cycle in glutamine metabolism for *de novo* synthesis of non-essential fatty acids [19]. Recent studies have shown that blocking key enzymes of fatty acid synthesis, such as fatty acid synthase (FASN) and ELOVL fatty acid elongase 2 (ELOVL2), suppressed glioma tumor growth in GBM xenograft models, further suggesting the importance of fatty acids for glioma growth and survival [27]. In contrast, other studies have linked fatty acid oxidation rather than its synthesis to the presence of “slow cycling” GBM progenitor cells that drive GBM growth and recurrence after initial therapy [28]. Subsequent studies on GBM neurospheres and their slow-growing progenitor cells revealed high level of fatty acid-binding protein 7 (FABP7), a lipid chaperone that mediates fatty acid uptake and oxidation, while its targeted inhibition suppressed GBM cells growth and invasiveness *in vitro* and *in vivo* [29,30]. A similar observation was made *in vitro* from primary cell cultures of human GBM and in tumorigenic intracranial GBM mice model [31]. These findings suggest that owing to the heterogeneous nature of GBM in regulating lipid metabolism, alternative promising combinatorial therapies may be required to improve the clinical outcome in patients with brain tumors.

### 2.4. Nucleotides

Nucleotides are signaling molecules necessary for the transmission of cellular energy sources (ATP and GTP) and various other metabolic processes. A previous study found elevated d*e novo* purine synthesis in GBM tumors, which promoted the maintenance of brain tumor-initiating cells *via* constitutive upregulation of *c*-MYC activity [32]. GBM cells also rely on the purine salvage pathway. Indeed, the ability to scavenge hypoxanthine, the most abundant purine in the cerebrospinal fluid (CSF), may explain GBM resistance to anti-folate therapy [33]. Furthermore, combinatorial targeting of both *de novo* and salvage pathways of nucleotide synthesis in brain tumor-initiating cells has been reported to inhibit glioblastoma progression [34]. Based on these observations, a clinical trial of combination therapy with gemcitabine and radiation in high-grade GBM patients showed reasonable safety and promising clinical outcomes [35]. In contrast, several resistance mechanisms that are independent of purine salvage have been identified in GBM, which affect tumor evolution and physiology and pose clinical challenges [36]. Notably, nuclear magnetic scans using the radiolabeled tracer ^18^F-fluorothymidine (^18^F-FLT) revealed an increase in FLT radiotracer in GBM tumor tissues compared to the adjacent normal brain tissues [37]. As nucleotide metabolism is a well-studied and well-established therapeutic target, gaining a better understanding of altered nucleotide metabolism in GBM can lead to therapeutic advances.

## 3. Brain metastases and their metabolic adaptation

Brain metastasis (BrM) is the most common malignancy that affects the central nervous system (CNS) and principally involves brain parenchyma and leptomeninges. Despite the use of advanced therapies, BrM outcomes are frequently poor due to high morbidity and mortality, with median survival of less than one year [2]. An estimated 15–30% of cancer patients develop brain metastasis, particularly in patients with lung (39–56%), breast (13–30%), and colorectal cancer (3–8%), and melanoma (10–11%), [2,3] (Table 1). However, actual incidence rate is likely to be higher owing to advanced imaging techniques and improved systemic therapies, which render selective pressure by targeting aggressive, brain-penetrating phenotypes [2]. Interestingly, the brain microenvironment differs significantly from extra-cranial lesion microenvironments owing to its anatomical location and the presence of unique cell types with distinct metabolic constraints and immune environment. This imposes a distinct selective pressure on colonizing tumor cells, which shapes the metastatic process and therapeutic response. Therefore, it will be interesting to decipher the underlying mechanism(s) driving brain metastases to develop preventive therapeutic strategies that will provide further insight in context of primary brain tumors with similar challenges. Accordingly, recent studies have indicated metabolic processes as determinants of brain metastases. Jin et al. identified SREBP1 as a key factor that allows cancer cells to metastasize to the brain. Furthermore, Ferraro et al. demonstrated that cancer cells use fatty acid synthesis to survive in a lipid-deprived brain microenvironment [38,39]. These exciting findings are critical for understanding how metabolic reprogramming promotes metastasis, which could open new therapeutic avenues to improve clinical outcomes in patients with brain metastases.

Despite contributing to only 2% of the total body weight, energy demand of brain is the highest among all organs, consuming one-fifth of the body’s glucose-derived energy [40]. Furthermore, in response to varying metabolite availability, starvation, or changing neuronal activity, brain has a unique ability to rewire its metabolism [41]. According to current studies, metabolic adaptations required for brain metastases are primarily associated with brain-specific metabolites and the interactions between brain cells and tumor cells. A previous study using a proteomics approach showed increased expression of metabolic enzymes involved in glycolysis, TCA cycle, OXPHOS, pentose phosphate pathway (PPP), and glutathione system in brain metastasizing cancer cells to obtain energy *via* oxidative metabolism of glucose [42]. However, in response to oxidative stress, brain metastasizing cancer cells activate alternative glucose metabolic pathways to maintain redox homeostasis for survival and growth [42]. Similarly, another study used NMR spectroscopy to compare related metabolites between gliomas and brain metastasis by infusing ^13^C-labeled glucose during surgical resection and found that both gliomas and brain metastasis showed similar metabolic profiles [43]. In addition to glucose metabolism, lipid metabolism has been shown to have a significant effect on brain metastases. Cordero et al., in well-designed *in vitro* and *in vivo* experiments, demonstrated that FABP family members could support the glycolytic phenotype and lipid droplet storage for survival of cancer cells during their initial adaptation to the brain microenvironment [44]. Furthermore, Mashimo et al. demonstrated that by increasing the activity of acetyl-CoA synthetase enzyme 2 (ACSS2), brain metastasizing cancer cells could fuel the TCA cycle by converting acetate to acetyl-CoA [17]. Brain metastatic cancer cells displayed remarkable metabolic flexibility by oxidizing branched-chain amino acids (BCAAs) and glutamine to survive and proliferate in the absence of glucose, and thus use them as an alternative fuel source [45]. Importantly, glutamine and BCAAs (such as leucine), are part of the astroglial-neuronal nitrogen shuttle and vital for maintaining brain homeostasis, which serve as an important energy source for metastatic cancer cells [19]. These findings suggest that cancer cells metastasizing to brain, just like the brain cells, are extremely flexible in their metabolic ability to utilize different energy sources for their survival in a non-conducive brain microenvironment.

Emerging evidence suggest that primary tumors play a role in creating microenvironment in distant organs for future metastasis. These preconditioned microenvironments are known as “pre-metastatic niche.” However, the significance of metabolic priming within premetastatic niche in facilitating metastatic cancer cell adaptation to these sites remains unknown. Importantly, a recent brain metastasis study found that tumor cell-secreted exosomal miR-122 reprogrammed glucose metabolism in resident cells within the pre-metastatic niche in pyruvate kinase (PKM)/glucose transporter 1 (GLUT1)-dependent manner, which accommodates their nutrient requirements and contributes permissive growth during brain metastasis [46]. Further research on metabolic dependencies of metastasizing cancer cells in the pre-metastatic niche, which arise from primary cancer cells with high glycolytic activity or deriving energy from other nutrient sources to target and impose metabolic constraints during disease progression, will be of great clinical significance.

## 4. Metabolic interactions between brain cells and metastatic cancer cells

Metastatic progression is influenced by both brain microenvironment and intrinsic properties of metastatic tumor cells. However, because of the high level of heterogeneity among cell populations in the brain microenvironment, cancer cells can exploit the properties of these cells for survival and maximal growth at the metastatic site. Neurons, the principal components of the CNS, can communicate with cancer cells *via* neurotransmitters, such as gamma-aminobutyrate (GABA), to promote their colonization during brain metastases [47]. A previous study on brain tumor tissues from HER2-positive and triple-negative breast carcinomas revealed that brain metastatic cancer cells express GABAergic features, including GABAA receptors, GABA transporters, GABA transaminase, and glutamate decarboxylase, which help metastasizing cancer cells to activate GABA catabolism to increase their NADH levels to gain proliferative advantage [47]. Furthermore, a recent study showed that activation of glutamate stimulates the GluN2B-mediated *N*-methyl-d-aspartate receptor (NMDAR) signaling axis in brain metastasizing cancer cells *via* functional interaction with neurons, creating a supportive TME in orchestrating brain metastasis [48]. However, it is yet to be determined whether brain metastasis from other primary tumors similarly activate and utilize NMDAR-dependent signaling of glutamatergic neurons for colonization. Interestingly, glutamate is the major excitatory neurotransmitter in the CNS, and plays an important role in the formation and plasticity of glutaminergic synapses (*N*-methyl-d-aspartate; NMDA) through NMDAR receptor [49]. It is also involved in brain homeostasis along with astrocytes, supporting glial cells that maintain the balance between extracellular ionic environment and pH, and extracellular glutamate uptake and release, and provide metabolic substrates for neurons [50]. Importantly, glutamine and BCAAs (such as leucine), are abundant in brain microenvironment and are vital for maintaining brain homeostasis. Astroglial-neuronal nitrogen shuttling responds efficiently to subtle changes in glutamate levels to sustain neurotransmission, and thus constitutes an important energy source that can be readily exploited by metastasizing cancer cells for successful colonization [48]. For example, malignant brain tumor cells have been observed to utilize astrocyte gap-junctional network to transfer the second messenger cGAMP to activate and produce inflammatory factors, interferon alpha (IFNα) and tumor necrosis factor (TNF) *via* the STING signaling pathway in astrocytes. This, in turn, activates the STAT1 and NF-κB pathways, resulting in brain metastasis and drug resistance [51]. Intriguingly, brain metastatic cells were found to have a tumor metabolic phenotype, similar to that of CSF tumor cells owing to increased PTGS2 expression, which assisted cancer cells in passing through tight capillary connections during tumor colonization [52]. Furthermore, myelin-producing oligodendrocytes, another prominent glial cell within the CNS, receive synaptic inputs from neurons and participate in metabolic homeostasis of brain microenvironment [53]. These findings suggest that CNS-bearing resident cells are closely related to metastasizing cancer cells that are metabolically flexible in utilizing different fuels for their survival in a hostile environment during brain colonization. However, further research into the metabolic interactions between malignant tumor cells and brain cells during parenchymal brain metastasis and leptomeningeal metastasis require further investigation.

## 5. Nutrient availability determines metabolic dependence during metastasis

Differential nutrient and metabolite availability are essential factors governing metabolic plasticity during metastasis. Current metabolic research is majorly focused on tumor migration and invasion, an early stage of metastasis, with only a few studies focusing on metabolic classification of nutrients in tumor cells in circulation and at late metastatic stage. Here, we discuss the metabolic aspects of a few reported nutrients mainly pyruvate, lactate, glutamine, and fatty acids, that regulate tumor cell colonization at metastatic sites.

### 5.1. Pyruvate and lactate metabolism

Lactate dehydrogenase (LDH) is a primary metabolic enzyme that catalyzes interconversion of pyruvate to lactate and *vice versa* in TME. Furthermore, pyruvate and, consequently, lactate can be produced from glutamine and other amino acids, oxidized in the mitochondria to acetyl-CoA by pyruvate dehydrogenase (PDH), or converted to oxaloacetate by pyruvate carboxylase (PC), which replenishes the TCA cycle to support biomass production (anaplerosis). Interestingly, to adapt to the new environment for proliferation and survival, tumor cells at the primary site evolve for uptake of both lactate and pyruvate nutrients from the microenvironment. Contrastingly, during tumor progression, colonizing cancer cells at the secondary site are likely exposed to different types and/or levels of nutrients to create an accommodating metastatic niche. For example, pyruvate concentrations are significantly enriched in lung microenvironment during metastasis, which promotes higher PC-dependent anaplerosis than in primary cancers across several cancer types [54]. Thus, it is plausible that metastasizing cancer cells utilize pyruvate during early colonization. Similar evidence of proline metabolism has been reported *in vivo* for metastasizing cancers [55]. However, the mechanisms by which nutrients such as lactate, pyruvate, and proline, affect cancer cell colonization in the pre-metastatic niche is still unknown.

### 5.2. Glutamine metabolism

Glutamine is the most abundant circulating amino acid in plasma and tissues. Many cancer cell types primarily utilize glutamine for survival and growth and show altered glutamine metabolism. Glutamine is a non-essential amino acid that serves as a source of reduced nitrogen for biosynthetic reactions and carbon for anaplerosis, and can be fully oxidized to produce ATP (glutaminolysis). A recent study observed that glutamine transporter SLC1A5 (ASCT2) that mediates and metabolizes ^18^F-FGln uptake was expressed at low levels in normal brain tissues but at much higher levels in gliomas [23], and facilitates clear tumor delineation, indicating that glutamine is a vital fuel for cancer cell colonization [23]. Recent studies have also shown that both ^13^C-labeled glutamine uptake and *de novo* glutamine synthesis upregulated glutamine metabolism and contributed to therapy resistance in GBM cell lines, pre-clinical models, and clinical samples [21]. Accordingly, treatment of systemic metastasis in a pre-clinical model of brain tumor using glutamine analog 6-diazo-5-oxo-L-norleucine (DON) resulted in decreased metastases to distant organs [56], implying the importance of metabolic therapies to target glutamine metabolism in tumor metastasis treatment.

### 5.3. Fatty acid metabolism

Fatty acids are vital fuels for anabolic and catabolic processes. They are synthesized *de novo* but are mostly taken up from extracellular space [57]. Uptake of essential fatty acids, such as linoleic acid, is necessary for synthesis of most polyunsaturated fatty acids, which are frequently desaturated to monounsaturated fatty acids, important building blocks of membrane lipids. Thus, desaturation of fatty acids is detrimental to the survival of malignant tumor cells in metabolically compromised environment [58]. Furthermore, many fatty acids are oxidized in mitochondria to acetyl-CoA, whereas few long-chain and branched-chain fatty acids are oxidized in peroxisomes. Although little is known about the availability of fatty acids at target sites of metastases relative to primary tumors, there is ample evidence that fatty acid uptake and metabolism can boost the nesting of metastasizing cancer cells in multiple organs. Metastases has also been linked to *de novo* fatty acid synthesis. For example, targeting CD36, which plays a key role in fatty acid transport, had minor effects on primary tumor growth but significantly impaired metastasis [59]. Moreover, recent studies have shown that upregulation of fatty acid synthesis is required for metastasis in lipid-poor environment in the brain. Ferraro et al. demonstrated that HER2-positive breast cancer cells can synthesize fatty acids from glucose in a lipid-deprived brain microenvironment, thereby enabling metastasis [38]. Furthermore, a recent study by Jin et al. identified SREBP1 as a key factor governing cancer cells ability to metastasize to the brain. Notably, fatty acid dependency was not only constrained to breast cancer, but also observed across a large compendium of 500 cancer cell lines and 21 solid tumor types [39]. Additionally, in a recent study, altered lipid metabolism in cancer cells has been observed in acute lymphoblastic leukemia (ALL), as a part of metabolic adaptation to brain microenvironment during their migration. CNS-derived ALL cells showed upregulation of many genes encoding fatty acid synthesis-related molecules compared to the peripheral cells [60]. These studies maximize our understanding of the brain TME and reveal the importance of fatty acid and lipid metabolism in cancer cells metastasizing to the brain by combining high-throughput multi-omics datasets from genome, proteome, transcriptome, and metabolome-based approaches. Collectively, these findings will have a significant translational impact on brain metastases that are treatment-refractory.

### 5.4. Additional metabolic repertoire

The studies described above infer different metabolic reprogramming in cancer cells that can potentially be targeted during transition through metastatic cascade. Notably, there are additional metabolic repertoire, including acetate, serine, proline, and asparagine, which have been evaluated for a limited number of metastatic steps and act as alternative fuels and nutrients in various cancers [17,61,62]. Further studies on these factors in brain metastases may reveal novel targetable pathways.

## 6. Immune contexture and metabolic plasticity in brain metastasis

**Fig. 4.**
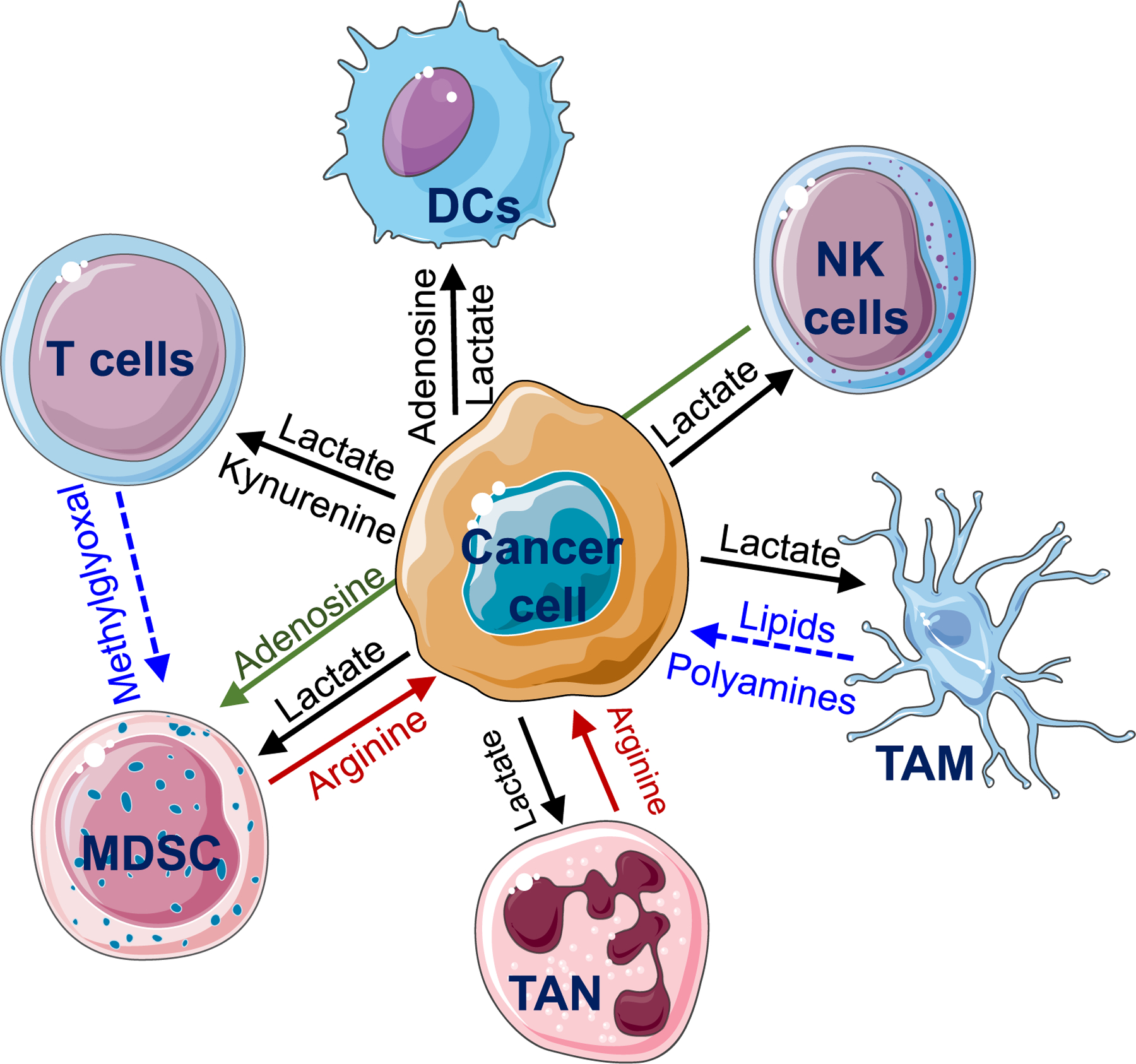
Metabolic crosstalk between cancer and immune cells in metastatic TME.

Brain is comprised of heterogeneous TME, both in its architecture and in its evolution from early to late disease and its architecture, with differences detected within tumors, as well as among cancer-bearing individuals with similar diagnosis, non-neoplastic cells types, and cell states, and among individual tumor cell clones. The immune context of brain TME comprises distinct cell types [63] and emerging evidence suggest that metabolism is a key regulator of immune phenotype and function [64]. Therefore, it is crucial to understand how metabolic alterations in these cells control their pro or anti-tumor properties during brain metastasis (Fig. 4).

Immune evasion has been recognized as an emerging hallmark in cancer progression [65]. In addition to immune escape, immune cells are adapted/captured by cancer cells to support their growth during metastatic progression. Importantly, majority of metastatic cells entering brain fail to grow and form macro-metastases [66], indicating the presence of antagonistic processes that prevent colonization during establishment. Compelling evidence also showed that brain-resident immune and stromal cells can also occasionally contribute to a favorable microenvironment, suggesting new mechanism to brain metastases [51,67]. Interestingly, studies show that resident microglia can selectively inhibit or promote tumor cell growth during colonization by upregulating tumoricidal (nitric oxide) or growth-supporting (neurotrophin-3) factors, respectively [68,69]. This suggests that during the progression of brain metastases, these aspects largely depend on the specific tumor type and its associated microenvironment. Additionally, microglia have been shown to upregulate interleukin (IL)-6 and CXCL5/8 chemokines during brain metastasis, exerting immunosuppressive effect and resistance to therapy [63,70]. Similarly, in metastatic niche, astrocytes that normally protect brain from cancer cells facilitate entry and early brain colonization by expressing high levels of IL-1β, IFNα, and TNF *via* Notch and cGAMP-mediated STING pathways [51, 71]. Furthermore, extracellular matrix (ECM) degrading factors, such as heparanase and matrix metalloproteinases (MMPs), produced by reactive astrocytes provide physical and chemical cues that aid cancer cell migration and invasion during early stage of brain metastases [72]. Importantly, by expressing inflammatory chemokine S100A8/A9, the active influx of myeloid cells (CD11b+Gr1+) in brain facilitated the entry and survival of colonizing metastatic tumor cells [73]. It has been reported that high CCL9 expression in myeloid cells contributes to pre-metastatic niche formation and promotes tumor cell survival in TGF-β-dependent manner [74]. Moreover, mast cells and natural killer (NK) cells have been shown to participate in intra-tumoral immune cell population in brain TME and contribute towards development and survival of brain metastases [75,76]. Overall, immune system plays a critical role in multiple stages of brain metastasis; therefore, gaining a better understanding of the metabolism of TME could lead to discovery of new therapeutic approaches against brain metastasis. The metabolic aspects of immune cells and their implications in brain tumor and metastasis are discussed here.

### 6.1. Tumor-associated macrophage/microglia

Tumor-associated macrophages/microglia (TAMs) are important immune sentinels in brain TME, and are associated with immune suppression and expansion during tumor progression. TAMs exhibit metabolic plasticity and close crosstalk with tumor cells, making them attractive therapeutic targets for modulating tumor-specific immunity in the brain. In glioma and brain metastases, TAMs outnumber other immune cells such as T-cells, NK cells, and neutrophils. The oncometabolite 2-hydroxyglutarate, produced by mutant IDH1 and IDH2-encoding enzymes, influences TAM composition in high-grade gliomas [63]. TAMs also utilize glycolysis and PPP to meet their bioenergetic requirements by changing their polarity in response to altered metabolic profile of TME [77,78]. According to recent studies, TAMs are forced to compete with tumor cells for nutrients, particularly glucose, in a nutrient-deprived TME and undergo similar changes as tumor cells for their glucose metabolism. Activated TAMs utilize aerobic glycolysis, which contributes to their functional metabolic reprogramming [79]. TAMs also exhibit changes in their lipid metabolism over glucose metabolism in the TME to adapt to environmental alterations associated with functional reprogramming by increasing fatty acid storage, uptake, and biosynthesis [80]. Furthermore, pro-tumorigenic TAMs exhibit increased glutamine-related metabolism and activated UDP-GlcNA pathway to maintain M2 polarization [78]. However, the underlying mechanism that defines TAM’s intrinsic relationship with tumor is not fully understood. Therefore, additional extensive research involving comprehensive metabolomic mapping is required to understand the metabolic adaptation that supports TAM phenotype.

### 6.2. Dendritic cells

Dendritic cells (DCs) are the most potent antigen-presenting cells (APCs), capable of internalizing, processing, and presenting antigens to tumor-reactive T-cells in lymph nodes and various tissues, including brain [81]. In human GBM, at least five DC subsets have been identified: conventional DC1 (cDC1), cDC2, migratory DCs, pre-DCs, and plasmacytoid DCs (pDCs) [63]; three of these (cDC1s, cDC2s, and pDCs) have recently been identified during brain metastases [76] as well as in human and mouse choroid plexus and meninges [82]. In the resting state, DCs metabolically utilize OXPHOS, however, upon activation, they switch to glycolysis. Intriguingly, glioma cells use glycine-*N*-methyl transferase to convert glycine to sarcosine that competes with DCs for glycine uptake *via* glycine transporter type-1 [83]. Furthermore, inhibition of glycolysis or LDHA showed repression of glioma tissues infiltrated by malignantly transformed DCs and immune tolerance in a recent GBM pre-clinical study [84]. Importantly, photodynamic therapy-induced lipid peroxidation and ferroptosis mediated release of damage-associated molecular patterns (DAMPs) factors such as calreticulin, high mobility group protein B1, and ATP by dying glioma cells promote DC maturation and activation [85]. Furthermore, restoring DC anti-glioma functions through glioma cell metabolism dominance is critical for antigen presentation and recognition abilities, which can authentically exert DC vaccine efficacy on gliomas. Further research on the relationship between glioma cell metabolism and DC recruitment is needed to better understand the immune microenvironment of brain lesions.

### 6.3. T-cells

Metabolic reprogramming is inextricably linked to T-cell survival and proliferation. Recent advances in the field of immunometabolism have identified distinct metabolic pathways, such as glycolysis, OXPHOS, and FAO (fatty-acid oxidation), which play critical roles in governing T-cell activation and differentiation, and their modulation can differentially affect the growth of T-cell subsets [86]. In addition to being metabolically diverse, T-cell subsets, such as cytotoxic T lymphocytes (CD8^+^, CTLs) and regulatory T-lymphocytes (CD4^+^CD25^+^FOXP3^+^, Tregs), form an active lineage of glioma-infiltrating lymphocytes that regulate immune response and peripheral tolerance [87]. A recent pre-clinical study found that depleting the amino acid stress-sensing pathway in hypoxic niduses impaired CD8^+^ T-cell activation and function in GBM, resulting in decreased viability and exhaustion of T-cells [88]. In contrast, another study demonstrated that Tregs in hypoxic TME are unaffected by glucose deprivation and primarily rely on fatty acids in response to glioma-secreted cytokine CCL22, which regulates their migratory capacity in an HIF-1-dependent manner [89]. Intriguingly, inhibition of lactate production in glioma cells, which drives Treg activation and infiltration, contributed to impaired Treg-mediated immune suppression in malignant gliomas [90]. Notably, fatty acid metabolism, including *de novo* fatty acid synthesis and FAO, plays a key role in regulating and promoting effector T-cell differentiation and Treg cell development [91]. Overall, these findings suggest that TME metabolically modulates adaptive immune response during glioma progression. Therefore, a comprehensive understanding of metabolic reprogramming in T-cells in relation to immunological diseases is essential for future clinical drug development.

Chimeric antigen receptor (CAR)-T cell-based therapies have shown significant efficacy and potential in clinical trials involving hematological malignancies [92]. However, this therapy has been unsuccessful in clinical trials involving difficult-to-treat cancers such as GBM that showed consistent tumor recurrence [93]. This is because of immunosuppressive TME and persistence of a dynamic metabolic state in patients CAR-T cells, both of which limit therapeutic efficacy and influence insufficient trafficking of dysfunctional and exhausted CAR-T cells [94]. Interestingly, CAR signaling domains within different T-cell subtypes use distinct metabolic pathways associated with their functions to mediate metabolic reprogramming by modifying bioenergetics and biogenesis. Furthermore, emerging strategies for enhancing the metabolic function of adoptively transferred CAR-T cells are actively being investigated to improve their efficacy against solid tumors. This includes reengineering CAR-T cells to express arginine resynthesis enzymes, such as argininosuccinate synthase and ornithine transcarbamylase, which are essential for supporting effector function in the arginine-low TME [95]. Similarly, catalase-secreting genetically modified CAR-T cells have been developed to protect them from ROS damage, as well as from unmodified bystander effect of unmodified immune cells, thereby improving their effector function and therapy outcomes [96]. Furthermore, study on the mechanistic effect of lactate on CAR-T cell metabolism and function has shown that inhibiting LDH has a direct effect on boosting CAR-T memory and enhancing downstream anti-tumor immunity [97]. Geltink et al. recently demonstrated the potential use of transient glucose restriction to metabolically enhance CAR-T cell effector and anti-tumor functions, thereby promoting their efficacy for adoptive cell therapy [94]. Overall, these findings suggest that reprogramming CAR-T cell metabolism is an appealing approach that could lead to improved anti-tumor functions, increased persistence, and superior adaptation to the nutrient-depleted TME.

### 6.4. Myeloid-derived suppressor cells

Myeloid-derived suppressor cells (MDSCs) are a heterogeneous population of innate immune cells that exhibit potent immunosuppressive activity on T-cells [98]. Intra-tumoral and systemic blood density of MDSCs significantly affected glioma progression and correlated with unfavorable prognosis and tumor-grade-dependent MDSCs frequency in glioma patients [99]. Emerging evidence have shown that inhibition of metabolic reprogramming induced by glutamine in tumor cells caused drastic reduction in MDSCs recruitment to the tumor bed, thereby influencing tumor cell growth and metastasis [100]. MDSC generation, infiltration, and immunosuppressive phenotype acquisition are generally regulated by glioma cell metabolism, which primarily relies on unsaturated fatty acids and amino acids through two mechanisms: (1) depletion of essential amino acids to T-cells, and (2) oxidative stress generation by reactive species. Recent studies have revealed that tumor-associated MDSCs of both monocytic and granulocytic origin prefer FAO than glycolysis as their primary energy source, with increased mitochondrial biogenesis and oxygen consumption rate, metabolic reprogramming, and subsequent secretion of arginase, nitric oxide, and peroxynitrite, which inhibit CD8^+^ T-cells [64,101]. Recently, lectin-type oxidized LDL receptor 1 (LOX-1) was reported to be overexpressed in polymorphonuclear-MDSCs, which is associated with a subpopulation of ER-stressed, immunosuppressive MDSCs in cancer patients for lipid uptake, providing further evidence for FAO activation in human MDSCs [102]. These findings suggest that tumor-associated MDSCs prefer lipids or fatty acids as an additional energy source to reprogram their metabolic pathways and adapt to thrive in low oxygen and glucose TME. However, studies on MDSC metabolic alterations, such as fatty acid and glycolysis utilization, remain unclear in various models of glioblastoma and brain metastases.

### 6.5. Neutrophils

Neutrophils are the most abundant circulating granulocytes and constitute critical immune sentinels in the TME. Tumor-associated neutrophils are observed in patients with IDH wild-type (IDHwt) and IDH mutant (IDHmut) gliomas [63] and brain metastases [103]. Recently, neutrophils were found to trigger ferroptosis and promote tumor progression in a xenograft mouse model of glioblastoma [104] and were negatively associated with advanced disease and poor survival in GBM patients [105]. Metabolically, neutrophils predominantly utilize aerobic glycolysis and PPP as their main source of energy metabolism despite possessing abundant mitochondria, which are generally used for maintaining redox balance essential for their survival. In addition, the anabolic metabolism of PGE2 by COX-2 expressing glioma cells increased neutrophil recruitment by upregulating IL-1β and CXCL1 expression [106]. Neutrophil extracellular traps (NETs) are another important structure of DNA and histones with anti-bacterial effect that switch their metabolism from glycolysis to PPP during formation of NETs, essential for trapping and killing bacteria [107,108]. Collectively, these events indicate how neutrophils undergo metabolic reprogramming in response to various environmental cues during tumor progression. Future mechanistic understanding of how neutrophils regulate distant metastases in the context of metabolism is of key clinical importance.

### 6.6. Natural killer cells

Natural killer (NK) cells are effector lymphocytes of the innate immune system, which play an important role in mediating anti-tumor and anti-viral activities. Recent single-cell mapping of human brain metastases identified tumor-associated NK cells as immature, highly cytotoxic, innate lymphoid-like cells co-clustered with CD8^+^ T cells in the CSF [76]. These cells play an intriguing role in brain tumor progression and heterogeneity by infiltrating pilocytic astrocytoma and metastatic brain neoplasms [109]. Furthermore, activated NK cells robustly undergo metabolic reprogramming to support rapid proliferation and effector functions [110]. Metabolic studies of mouse or human NK cells have shown substantial increase in the rate of OXPHOS and glycolysis, followed by an increased mitochondrial mass during chronic stimulation with various pro-inflammatory cytokines [110,111]. This indicates one of the underlying mechanisms by which activated NK cells upregulate key signaling pathways and mediate metabolic responses. Moreover, it has been recently demonstrated that TGF-β signaling can inhibit NK cell metabolism, including OXPHOS and glycolytic, and respiratory capacity in an IL-2 dependent manner, resulting in impaired immune response [112]. Therefore, a mechanistic understanding of how TME influences NK cell metabolic responses and contributes to the dysfunctional state during tumor progression will be useful for developing novel anti-cancer therapies.

## 7. Unravelling metabolic dependencies in brain tumor

Metabolic reprogramming is an important hallmark of cancer and a critical target for the development of novel anti-cancer therapies. Non-cancer components of TME, comprising stromal and immune cells, compete for nutrient availability and play pivotal roles in tumor progression and maintenance. Furthermore, metabolic dependencies between the tumor and its microenvironment are a double-edged sword that fuels and supports growing tumor metabolic demands by regulating signaling pathways such as mTORC1 and AMPK *via* nutrient sensors for their survival during brain tumor and metastasis [113]. In this regard, strategies for targeting specific nutrients or metabolic byproducts in the TME may limit precise targeting of tumor cells. In contrast, interactions between oncogenic alterations and the TME highlight a potential therapeutic opportunity at an advanced stage of the disease to target metabolic dependencies in a tissue-specific manner. For example, ^18^F-FDG PET scans revealed EGFR-driven glucose utilization in a subset of GBM tumors, which exhibited decreased glucose metabolism after acute EGFR inhibition and classified as metabolic responders, indicating that EGFR-targeted treatment is a promising metabolic therapy [114]. Similarly, several evidence suggest that molecular therapies targeting metabolic vulnerabilities in IDH-mutated gliomas and acute myeloid leukemia have proven beneficial and emerged as promising therapeutic strategies for improving patient prognosis. Some of them are currently undergoing Phase-I clinical trials with promising results in patients with IDH-mutated glioma and leukemia [115,116].

Different aspects of the physical and biochemical environment of the tumor for sequestering and competing nutrient utilization with immune cells could represent a targetable liability that alters therapeutic responses. For more than 90 years, the ketogenic diet (KD) has been one of the most well-known therapeutic dietary options for treating drug-resistant seizures in children and adults with epilepsy [117,118]. The main source of energy in KD is fat but it can be ketones if carbohydrate intake is reduced during ketosis. Certain GBMs lack the metabolic flexibility to utilize ketones as an alternative energy source unlike the normal brain [119]; therefore, depletion of glucose *via* a calorie-restricted KD may deprive GBMs of their dominant energy supply [120]. Despite the potential of KD as a dietary intervention to treat patients with highly glycolytic GBM and its implementation as a monotherapy or in conjunction with molecular therapy or radiation and chemotherapy in clinical settings, its practical application has always been invariably limited due to severe carbohydrate restriction. Furthermore, emerging evidence suggest that the tumor’s metabolic dependencies change as cancer progresses, particularly in the context of therapy resistance and metastases [121]. Future research that allows a comprehensive analysis of these emerging metabolic vulnerabilities will unravel the unique intricacies of multifaceted metabolic interactions to tailor therapeutic strategies for treating patients with brain tumors and metastases.

## 8. Targeting metabolism in the context of brain tumors

Despite the challenges associated with targeted metabolism in tumorigenesis, targeting metabolism represents a potential therapeutic perspective, as cancer cells have higher nutrient consumption than normal cells. Interestingly, enzymes, receptors, and proteins that participate in biochemical reactions during metabolism have numerous target-specific caveats that can be exploited pharmacologically, allowing the development of allosterically modified drugs to target enzymatic activity. Inhibition of key metabolic factors in brain tumor cells, on the other hand, is challenging, as developing neurons and glial cells are metabolically active, and their inhibition can lead to CNS toxicity. Importantly, many drugs fail to penetrate the protected brain due to the presence of formidable obstacles, the BBB, making the design of brain tumor-targeting therapies very challenging. Furthermore, the presence of tumor heterogeneity, tumor stem cells, and differences in the microenvironment exacerbate these difficulties, as the pathways associated with metabolic alterations in brain tumors are still unknown. By convergent activation of the RTK and PI3K/AKT/mTOR pathways, which regulate downstream signaling, more than 90% of GBMs show glucose and glutamine-specific metabolic alterations [122]. Nonetheless, focusing on tumor cell dynamics and similar metabolic traits, as well as their microenvironment, can help to improve beneficial effects against tumor growth and progression. This raises the possibility of treating brain tumors with combinations of conventional therapies by inhibiting multiple metabolic targets. ACSS2 [17], FASN [123], ELOVL2 [27] and acyl-CoA binding-protein (ACBP) [28] are examples of the key pathway enzymes for acetyl-CoA and fatty acid synthesis, which have been identified as potential drug targets for RTK- or EGFR-driven brain tumors. Consequently, GBM cells are highly dependent on astrocyte cholesterol synthesis *via* PI3K/SREBP-1-dependent upregulation of LDLR, suggesting that LXR–cholesterol axis may generate an actionable vulnerability in gliomas [26]. Furthermore, both brain tumors and metastases show upregulated glycolysis and OXPHOS [16,124] for which many potential drugs, including metformin and phenformin, that target bioenergetic pathways have been identified as therapeutic alternatives in the clinical setting [125–127]. These examples demonstrate the potential benefit of targeting metabolic liability and flexibility to treat brain thriving cancer cells.

## 9. Future directions

While metabolic targeting of primary cancers theoretically offers potential opportunities in the clinical setting, there has been growing interest in expanding our knowledge of how cancer cells harness metabolism during tumor progression and metastasis. Recent studies have highlighted the importance of metabolic plasticity and diversity in promoting tumor survival. Metabolic reprogramming is considered a dynamic and adaptive cancer hallmark that provides opportunities for cancer diagnosis, prognosis, and treatment. Over the past decade, several advances in metabolomic techniques have dramatically improved our understanding of the metabolic needs and vulnerabilities that evolve as tumor progresses from premalignant lesion to invasive metastatic tumor. Furthermore, because of their increased bioenergetic and biosynthetic demands to mitigate unfavorable oxidative stress for successful colonization at the metastatic site, metabolic plasticity governs the probability of cancer cell proliferation and survival through the metastatic process. Additionally, there is growing appreciation that the metabolism of non-cancer cell types in TME, including immune cells, can influence tumor progression and therapeutic response. Notably, there is keen interest in exploiting dietary interventions as a combinatorial therapy to target metabolism. The metabolic concept highlighted here discusses the emerging traits and vulnerabilities of cancer cells and the host microenvironment that supports their metastatic capacity at distant sites; however, an extensive research is needed to fully understand this mechanism. Finally, therapeutic targeting of metabolic reprogramming appears promising as a combinatorial approach for enhancing anti-cancer responses. Therefore, it is necessary to investigate the emerging metabolic vulnerabilities of brain tumors/metastases with a comprehensive mechanistic understanding to expedite basic research findings into clinical applications and to develop a therapeutic strategy to improve patient outcomes.

## Figures and Tables

**Table 1:** Brain metastasis incidence in various solid tumors.

Cancer Type	Brain metastasis incidence at initial diagnosis (%)	Brain metastasis among patient with metastatic disease (%)	Median survival after brain metastasis diagnosis (months)
Non Small	15–20	20–40	6
Cell Lung			
Cancer			
Small Cell	10–12	22	4
Lung			
Cancer			
Breast Cancer	14	13	10
Melanoma	10	28	4–6
Real Cancer	3–17	25	3–4
